# Antioxidant, Anti-Inflammatory, and Oral Bioavailability of Novel Sulfonamide Derivatives of Gallic Acid

**DOI:** 10.3390/antiox14040374

**Published:** 2025-03-21

**Authors:** Dania Alhyari, Nidal A. Qinna, Helen M. Sheldrake, Sriharsha Kantamneni, Bayan Y. Ghanem, Krzysztof J. Paluch

**Affiliations:** 1 School of Pharmacy and Medical Sciences, Faculty of Life Sciences, University of Bradford, Richmond Rd, Bradford BD7 1DP, UK; dhiary@uop.edu.jo (D.A.); k.j.paluch@bradford.ac.uk (K.J.P.); 2 Faculty of Pharmacy and Medical Sciences, University of Petra, P.O. Box 961343, Amman 11196, Jordan

**Keywords:** 3,4,5-TMBS, 3,4,5-THBS, cytochrome P450, COX-2 inhibition, pharmacokinetics, rat model

## Abstract

Gallic acid (GA) is known for its antioxidant and anti-inflammatory properties, yet its clinical potential is hindered due to poor oral bioavailability. This study investigates novel GA sulfonamide derivatives, 3,4,5-trimethoxybenzenesulfonamide (3,4,5-TMBS) and 3,4,5-trihydroxybenzenesulfonamide (3,4,5-THBS), and determines their antioxidant and anti-inflammatory activities and bioavailability. Antioxidant activity was evaluated using DPPH, FRAP, and ROS assays in human intestinal epithelial cells (HIEC-6). Protein denaturation and COX-2 inhibition were assayed to measure anti-inflammatory effects. 3,4,5-TMBS metabolism was assessed via CYP2D6, and pharmacokinetics were profiled in Sprague Dawley rats. GA and 3,4,5-THBS showed a three-fold increase in ROS scavenging activity at 1000 µM (96% for GA, 93% for 3,4,5-THBS). 3,4,5-TMBS and 3,4,5-THBS demonstrated significant anti-inflammatory activity when compared to ibuprofen at concentrations ≥100 nM (*p* < 0.05). 3,4,5-TMBS (50 µM) exhibited high COX-2 inhibition (*p* < 0.001) unlike GA (50 µM) which had a low COX-2 inhibition effect (*p* > 0.05), compared to ibuprofen. The percentage of 3,4,5-TMBS metabolism increased from 65% to 81% at 1500 µM (*p* < 0.05) when metabolized by CYP2D6. Pharmacokinetic studies revealed that 3,4,5-TMBS and 3,4,5-THBS had significantly higher C_max_ and longer half-lives than GA, with 3,4,5-TMBS showing a half-life of 7.17 ± 1.62 h, compared to 3.60 ± 0.94 h for GA (*p* < 0.05). 3,4,5-TMBS and 3,4,5-THBS demonstrated superior antioxidant and anti-inflammatory effects in HIEC-6 compared to GA, with enhanced bioavailability. These findings support the potential of 3,4,5-TMBS and 3,4,5-THBS as effective alternatives to GA for clinical applications.

## 1. Introduction

Oxidative stress, characterized by an imbalance between free radicals and scavengers, is a key contributor to cellular damage and the development of various diseases, including cardiovascular disorders, cancer, and neurodegenerative conditions [1,2]. Reactive oxygen species (ROS) can damage proteins, lipids, and DNA, leading to impaired cellular function and structural integrity. To counteract these effects, both synthetic and natural antioxidants have been extensively studied for their ability to neutralize free radicals and mitigate oxidative damage [3,4]. However, the potential toxicity and pro-oxidative effects of synthetic antioxidants have prompted researchers to explore natural alternatives, particularly phenolic compounds derived from plants [5,6]. Among natural antioxidants, gallic acid (GA), a polyphenolic compound, has gained considerable attention due to its potent antioxidant and anti-inflammatory properties [7]. GA is abundantly found in plants such as tea [8], coffee [9], sumac, hazelnuts [10], and various fruits including strawberries and raspberries [11]. Its chemical structure, 3,4,5-trihydroxybenzoic acid, features hydroxyl groups that enable it to scavenge free radicals, chelate metal ions, and inhibit lipid peroxidation [12]. These properties make GA a promising candidate for therapeutic applications in managing oxidative stress-related diseases.

**Figure 1:**
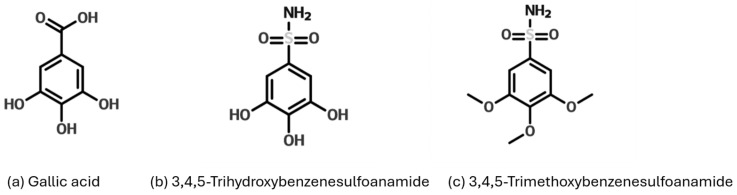
Structure of GA and gallic acid-based sulfonamides derivatives trihydroxybenzenesulfonamide (3,4,5-THBS) and trimethoxybenzenesulfonamide (3,4,5-TMBS).

The antioxidant activity of GA is further enhanced by its ability to stabilize free radicals through intramolecular hydrogen bonding and its low hydrogen bond dissociation enthalpy [12,13]. Additionally, its carboxylic group facilitates hydrogen donation, a critical mechanism in its radical-scavenging capacity. Beyond its antioxidant effects, GA exhibits significant anti-inflammatory activity by modulating key inflammatory pathways. It inhibits the release of pro-inflammatory cytokines such as TNF-α and IL-6, downregulates NF-κB and p38 MAPK signaling, and suppresses cyclooxygenase-2 (COX-2) and nitric oxide production [14,15,16]. These mechanisms highlight the potential of GA in treating inflammatory conditions, including post-stroke depression [17], and metabolic disorders [18]. Despite its therapeutic potential, the clinical application of GA is limited by its low oral bioavailability and rapid elimination from the bloodstream [19]. To address these limitations, researchers have focused on synthesizing GA derivatives, particularly sulfonamide-based compounds, which exhibit enhanced antioxidant and anti-inflammatory properties [20]. This study synthesizes and evaluates gallic acid-based sulfonamides, including trihydroxybenzenesulfonamide (3,4,5-THBS) and trimethoxybenzenesulfonamide (3,4,5-TMBS), as shown in Figure 1. The aim is to compare their antioxidant and anti-inflammatory properties with GA and determine whether these structural modifications improve GA’s bioavailability and therapeutic efficacy. The ultimate goal is to develop innovative natural-based therapies for diseases related to oxidative stress and inflammation.

## 2. Materials and Methods

### 2.1. Materials

All chemicals and reagents used in this study were of analytical grade. Ascorbic acid (99%), gallic acid (99.9%), copper chloride (99%), sodium nitrate (≥99%), sodium hydroxide (99.9%), sulfur dioxide (≥99%), CYP2D6, NADP+ (≥95%), and glucose-6-phosphate (≥95%) were sourced from Merck and Sigma Aldrich, located in Gillingham, UK. Additionally, 3,4,5-trihydroxybenzenesulfonamide (95%) was procured from Chemspace (Enamine Ltd., Kyiv, Ukraine), while DPPH (95%) was obtained from Santa Cruz Biotechnology in Dallas, TX, USA. Hydrochloric acid (37%), acetic acid (95%), acetone, iron(II) sulfate heptahydrate (99%), iron(III) chloride hexahydrate (97%), and 2,4,6-tri(2-pyridyl)-1,3,5-triazine were purchased from Fisher Scientific in Loughborough, UK. HPLC-grade solvents, including acetonitrile, dichloromethane, methanol, and ethanol, were also acquired from Fisher Scientific. Gibco Opti-MEM™ I Reduced Serum Medium, Gibco HEPES (1M), Glutamax, and Epidermal Growth Factor (EGF) were supplied by Fisher Scientific. Other reagents, such as trypsin, phosphate-buffered saline (PBS), DMSO, doxorubicin, Fetal Bovine Serum (FBS), and the Cell Counting Kit-8 (CCK-8), were purchased from Sigma Aldrich in St. Louis, MO, USA. The Cellular ROS Assay Kit, 2′,7′-dichlorofluorescin diacetate (DCFDA), and the cyclooxygenase activity assay kit were obtained from Abcam in Cambridge, UK. Laboratory equipment, including 6-well inserts with a 3.0 µm pore diameter (translucent and sterile), was sourced from Greiner Bio-One International in Frickenhausen, Germany. The Millicell^®^ ERS-2 Electrical Resistance device was purchased from Millipore in Burlington, MA, USA. Sterile 96-well plates with clear flat bottoms (black), 96-well plates, 15 mL and 50 mL centrifuge tubes, and 75 cm^3^ flasks were supplied by Sarstedt in Oberbergischer Kreis, Germany.

### 2.2. Methods

#### 2.2.1. Antioxidant DPPH Assay

The antioxidant properties of the compounds under study were examined using the 2,2-Diphenyl-1-picrylhydrazyl (DPPH) assay, following a well-established protocol [21]. A DPPH solution was prepared at a concentration of 0.1 mM in methanol. Then, 3 mL of this solution was mixed with 1 mL of the test compounds (GA, 3,4,5-THBS, or 3,4,5-TMBS), which were initially dissolved in methanol at concentrations ranging from 1 to 1000 μM (1, 3, 10, 30, 300, and 1000 μM). To ensure proper reaction, the mixtures were kept in the dark and incubated at room temperature for 30 min. After incubation, the absorbance of each sample was measured at 517 nm using a UV-VIS spectrophotometer. Gallic acid was used as the reference standard, and methanol served as the blank control.

The ability of the compounds to scavenge free radicals was determined by observing the reduction of DPPH at 517 nm, with lower absorbance values indicating higher antioxidant activity. The half-maximal inhibitory concentration (IC_50_), defined as the concentration of the compound needed to neutralize 50% of the DPPH radicals, was calculated from the dose–response curve. All experiments were performed in triplicate, and the results are reported as the mean of three independent trials. This method is widely recognized for its reliability and reproducibility in evaluating the antioxidant potential of chemical compounds.

#### 2.2.2. Ferric Reducing Antioxidant Power (FRAP)

The FRAP assay was conducted following an adapted procedure based on the method described by Benzie (1996) [22]. The FRAP reagent was prepared fresh and warmed to 25 °C before use. The reagent was made by combining 2.5 mL of a 10 mM TPTZ solution in 40 mM hydrochloric acid, 2.5 mL of a 20 mM FeCl_3_·6H_2_O solution, and 25 mL of a 0.3 M sodium acetate buffer adjusted to pH 3.6. For the assay, 100 μL of each sample was mixed with 3 mL of the freshly prepared FRAP reagent and incubated for 4 min. After incubation, the absorbance was recorded at 593 nm using a UV-VIS spectrophotometer, with methanol serving as the blank. A standard calibration curve was generated using ascorbic acid solutions at concentrations of 100, 200, 400, 800, and 1000 μM (R^2^ = 0.998). The FRAP values were calculated as μM of ascorbic acid equivalents based on the standard curve. The results presented are the mean of three independent experiments.

#### 2.2.3. Cell Viability Assay

Human intestinal epithelial cells (HIEC-6), originally obtained from the American Type Culture Collection (ATCC), were cultured in Opti-MEM™ I Reduced Serum Medium (Cat. No. 31985) supplemented with 20 mM HEPES, 10 mM L-glutamine, 10 ng/mL epidermal growth factor, and 4% fetal bovine serum (FBS) (New York, NY, USA). Cells were maintained in a humidified 5% CO_2_ incubator at 37 °C. Cell viability was assessed using the CCK-8 assay, a colorimetric method based on the reduction of water-soluble tetrazolium salts (WST-8) to a water-soluble formazan dye, with the resulting color intensity proportional to the number of viable cells [23]. Briefly, cells were harvested at approximately 80% confluence, detached using trypsin-EDTA, and centrifuged at 1000 rpm for 5 min. The cell pellet was resuspended in fresh medium, and the density was adjusted to 1 × 10^6^ cells/mL. A volume of 100 µL (1 × 10^5^ cells/well) was seeded into a 96-well plate, with one well containing the medium only as background control. After 24 h of incubation, the medium was replaced with fresh medium containing test compounds (GA, 3,4,5-THBS, and 3,4,5-TMBS) at concentrations ranging from 100 nM to 25 µM, followed by another 24 h incubation. Subsequently, 10 µL of CCK-8 reagent was added to each well, and the plate was incubated for 4 h. Absorbance was measured at 450 nm using a microplate reader, results are expressed as the mean ± standard deviation (SD).

In pharmacological and biochemical research, it is important to adjust concentrations to optimize the sensitivity and specificity of each assay. Varying concentrations between antioxidant assays such as DPPH and cytotoxicity tests were used in numerous studies like [24,25] as this strategy ensures that experimental conditions are carefully tailored to meet the unique demands of each method such as achieving an appropriate dynamic range of detection or maintaining biological relevance in the tested concentrations. In the current study, concentrations were determined through preliminary experiments and supported by the existing literature, ensuring the precise measurement of antioxidant activity and cytotoxicity while avoiding issues like interference or saturation. This approach is consistent with established methodologies in the field [24,25,26] and guarantees the reliability and robustness of the results across different assay types.

#### 2.2.4. 2′,7′-Dichlorofluorescein Diacetate (DCFDA) Assay in HIEC-6

The antioxidant DCFDA assay was conducted in HIEC-6 according to the protocol provided by the manufacturer Abcam (ab11385) and as mentioned by [27]. Cells were seeded at a cell density of 2.5 × 10^4^ cells/well in a clear bottom black 96-well microplate and allowed to adhere overnight at 37 °C in a CO_2_ incubator. Cells were washed and incubated in 100 µL of the diluted staining DCFDA solution, prepared by diluting 20 mM DCFDA in PBS, for 45 min at 37 °C in the dark Then, the DCFDA solution was removed and GA, 3,4,5-THBS, or 3,4,5-TMBS was added to the cells at different concentrations, in addition to doxorubicin, used as a positive control. Unstained cells were designated as blank. Fluorescence was recorded at Ex/Em = 485/535 nm.

#### 2.2.5. Protein Denaturation Assay

The anti-inflammatory potential of the compounds was evaluated using a protein denaturation assay, a well-established method to assess the capacity of test substances to inhibit protein denaturation, a key marker of inflammation [28,29]. The assay was performed as follows: The reaction mixture (5 mL) consisted of 0.2 mL of 0.1% Bovine Serum Albumin (BSA), 2.8 mL of phosphate-buffered saline (PBS, pH 6.4), and 2 mL of varying concentrations of reaction mixtures containing either gallic acid (GA), 3,4,5-THBS, or 3,4,5-TMBS (1–1000 nM). Distilled water served as the negative control, while ibuprofen, a known anti-inflammatory agent, was used as the positive control. The mixtures were incubated at 37 °C for 15 min to simulate physiological conditions, followed by heating at 70 °C for 10 min in a water bath to induce protein denaturation. After heating, the samples were allowed to cool to room temperature, and the turbidity of the solutions, indicative of protein precipitation, was measured spectrophotometrically at 660 nm. A reduction in turbidity corresponds to the inhibition of protein denaturation, reflecting the anti-inflammatory activity of the tested compounds.

#### 2.2.6. Cyclooxygenase (COX-2) Activity Assay

Cyclooxygenase (COX-2) activity assay was performed in HIEC-6 cells according to the manufacturer’s instructions (ab204699). Resorufin standards (0–20 pmol/well) were used to generate a calibration curve for quantifying sample concentrations. HIEC-6 cells were centrifuged at 500× *g* for 3 min and washed with PBS. The cell pellet was lysed in an ice-cold lysis buffer containing 10% Triton X-100 and a protease inhibitor cocktail in PBS for 5 min. The lysate was then centrifuged at 12,000× *g* for 3 min at 4 °C, and the supernatant was collected and kept on ice. Protein concentration in the supernatant was determined using a Bradford assay, with a bovine serum albumin standard curve (0–2000 µg/mL). A reaction mixture was prepared by combining cell lysate, COX-2 probes, COX-2 cofactor, and the test compounds GA, 3,4,5-THBS, 3,4,5-TMBS, or ibuprofen (positive control), along with a COX-2 inhibitor control. This mixture was added to a 96-well plate, and the reaction was initiated by adding arachidonic acid (COX-2 substrate) dissolved in NaOH solution. The enzymatic reaction produced fluorescent resorufin dye, which was measured at excitation/emission wavelengths of λEx/Em = 535/587 nm.

#### 2.2.7. Enzymatic Activity with Cytochrome P450 2D6 (CYP2D6)

The enzymatic activity of cytochrome P450 2D6 (CYP2D6) was evaluated following the CypExpress protocol (C4982, Sigma Aldrich, Darmstadt, Germany). The reaction buffer consisted of 5 mM glucose-6-phosphate (G6P), 2 mM nicotinamide adenine dinucleotide phosphate (NADP+), and 3,4,5-TMBS prepared in PBS at concentrations of 1000 and 1500 µM. Diltiazem was used as a positive control. The reaction was initiated by adding CYP2D6, and the mixture was vortexed for 5 min. The samples were then incubated at 37 °C with gentle shaking at 100 rpm for 4 h. The reaction was terminated by adding acetonitrile, followed by centrifugation at 800 rpm for 10 min. The supernatant was collected and analyzed using HPLC.

#### 2.2.8. Oral Bioavailability

Male Sprague Dawley rats, aged 7–9 weeks and weighing 220 ± 20 g on average, were used for bioavailability studies. The animals were housed in the Laboratory Animal Research Unit at the University of Petra Pharmaceutical Center under controlled conditions: a temperature of 23 ± 2 °C, humidity of 55–65%, and a 12 h light/dark cycle. Prior to the experiments, the rats underwent a 10-day acclimatization period with free access to standard food and water. All procedures complied with the University of Petra’s animal use guidelines, which adhere to the standards of the Federation of European Laboratory Animal Sciences Association (FELASA). The rats were divided into three groups (*n* = 6 per group) and administered suspensions of GA, 3,4,5-TMBS, or 3,4,5-THBS prepared in 5% carboxymethylcellulose. Each compound was administered orally at a dose of 100 mg/kg, based on previous studies [30]. Blood samples were collected from the retro-orbital plexus at 0.5, 1, 2, 3, 4, 6, 10, and 24 h post-administration. The samples were centrifuged at 5000 rpm for 10 min, and the serum was mixed with acetonitrile in a 1:3 ratio. After a second centrifugation at 5000 rpm for 15 min, the supernatant was collected for high-performance liquid chromatography (HPLC) analysis. HPLC analysis was conducted using a Shimadzu Class VP series system equipped with an LC-10AT VP pump, an SIL-10AD VP autosampler, and an SCL-10AVP system (Shimadzu, Kyoto, Japan). A C18 column (25 cm × 4.6 mm, 5 μm) was used, with a photodiode array detector set to a wavelength range of 200–400 nm. The mobile phase for TMBS and THBS consisted of a 50:50 (*v*/*v*) mixture of deionized water and acetonitrile containing 0.1% trifluoroacetic acid, flowing at 1 mL/min. For GA, the mobile phase was a 70:30 mixture of methanol and a water/sulfuric acid solution (100:2), also at a flow rate of 1 mL/min. The retention times for THBS and TMBS were 2.39 and 3.47 min, respectively, while GA eluted at 2.4 min, all detected at 254 nm. Results were analyzed using Lab Solution Software LC (version 5.3) and represent the average of three measurements.

## 3. Results

### 3.1. DPPH Assay

**Figure 2:**
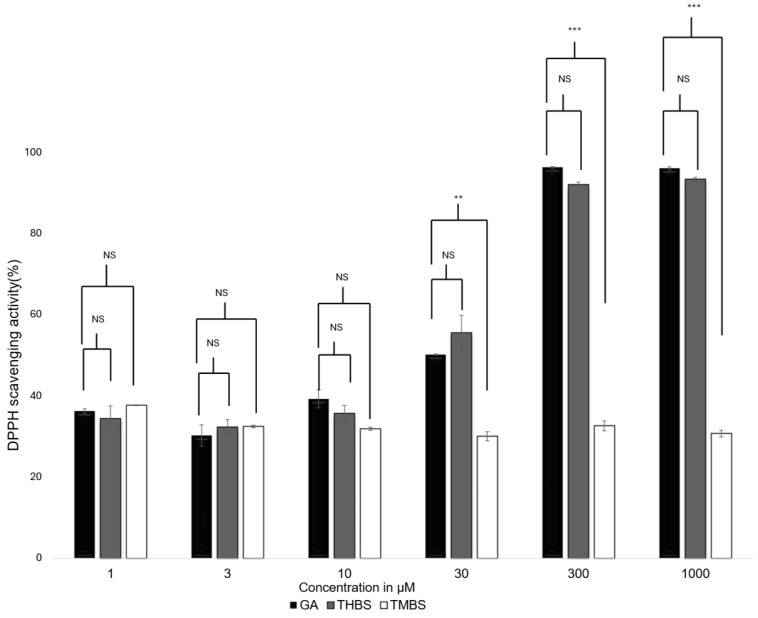
DPPH scavenging activity for GA (control), 3,4,5-THBS, and 3,4,5-TMBS at different concentrations. Also, 100% scavenging activity indicates the greatest antioxidant activity. (*n* = 3, mean ± SD). NS: non-significant; *p*-value > 0.05; ** *p* ≤ 0.01, *** *p* value ≤ 0.001.

The antioxidant activity of GA, 3,4,5-THB, and 3,4,5-TMBS were assessed using the 2,2-diphenyl-1-picrylhydrazyl (DPPH) radical scavenging assay. This method evaluates the ability of compounds to neutralize DPPH free radicals, with gallic acid serving as the reference standard. The scavenging activity of GA, 3,4,5-THB, and 3,4,5-TMB was tested at various concentrations, as shown in Figure 2. Both GA and THBS demonstrated a concentration-dependent increase in radical scavenging activity, with their activity exceeding 90% at 1000 µM. In contrast, TMBS showed minimal scavenging activity, with no significant change observed across the tested concentrations.

### 3.2. Ferric Reducing Antioxidant Power (FRAP) Assay

**Figure 3:**
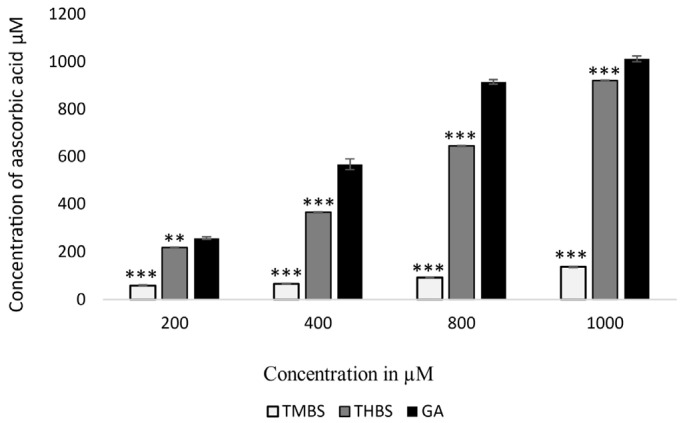
FRAP values of GA (control), 3,4,5-THBS, and 3,4,5-TMBS expressed in μM ascorbic acid equivalent. (*n* = 3, mean ± SD). ** *p* ≤ 0.01, *** *p* value ≤ 0.001.

The FRAP assay measures the antioxidant capacity of compounds by comparing them to ascorbic acid equivalents. In this study, both 3,4,5-TMBS and 3,4,5-THBS demonstrated lower antioxidant activity compared to GA, as illustrated in Figure 3. The FRAP values for GA and THBS were significantly higher than those for TMBS, indicating that GA and THBS have a greater ability to reduce Fe^3+^-TPTZ to the Fe^2+^ form, reflecting their superior antioxidant potential.

### 3.3. Cell Viability Assay

**Figure 4:**
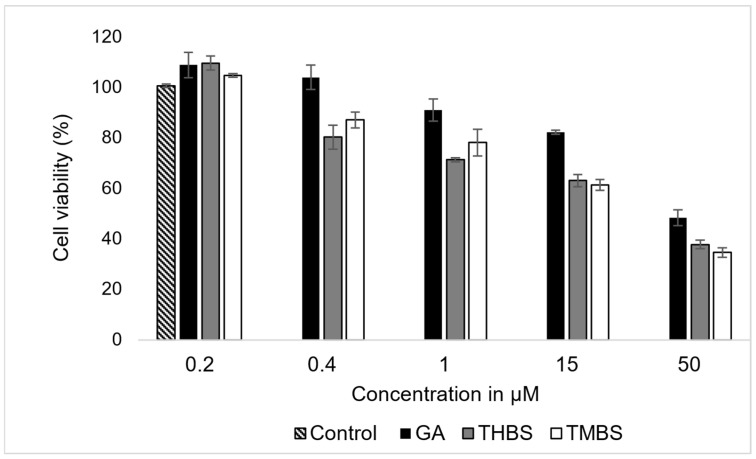
The effects of GA, 3,4,5-THBS, and 3,4,5-TMBS on HIEC-6 cell viability, as determined by cell count assay. Cells were treated with compound concentrations ranging from 0.2 to 500 µM (*n* = 3, mean ± SD).

The cytotoxic effects of GA, 3,4,5-THBS, and 3,4,5-TMBS on HIEC-6 cells were evaluated by assessing cell viability following a 24 h incubation period at concentrations ranging from 0.2 to 50 µM. The results, presented in Figure 4, demonstrate a concentration-dependent reduction in cell viability for all three compounds.

The data revealed a progressive decline in cell viability with increasing concentrations of GA, 3,4,5-THBS, and 3,4,5-TMBS. The IC_50_ for each compound was derived from the dose–response curves and is summarized in Table 1. GA exhibited an IC_50_ value approximate to 47 µM, while 3,4,5-THBS and 3,4,5-TMBS demonstrated lower IC_50_ values of 35 and 33 µM, respectively. These findings indicate that GA was less potent in reducing cell viability compared to 3,4,5-THBS and 3,4,5-TMBS, both of which displayed stronger cytotoxic effects at lower concentrations.

The results underscore the concentration-dependent cytotoxic activity of all three compounds, with cell viability inversely correlated with increasing compound concentrations. These findings provide critical insights into the differential cytotoxic profiles of GA, 3,4,5-THBS, and 3,4,5-TMBS in human intestinal epithelial cells.

### 3.4. Reactive Oxygen Species (ROS) Assay in Human Epithelial Cells

**Figure 5:**
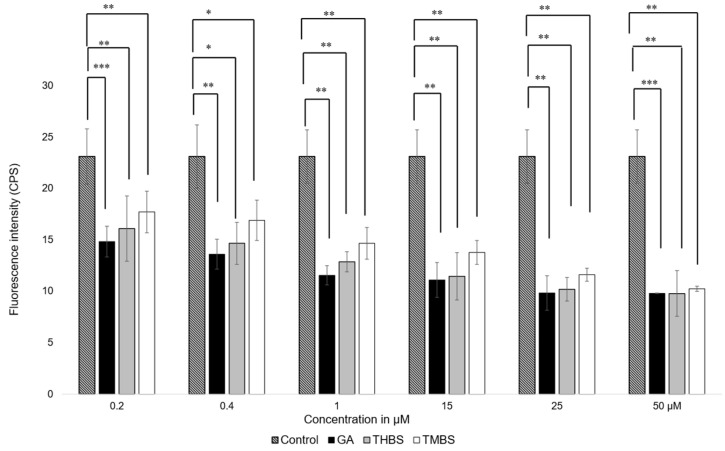
Fluorescence intensity measurements of ROS levels in HIEC-6 treated with GA, 3,4,5-THBS, and 3,4,5-TMBS (0.2–50 µM), as determined by the H2DCFDA assay. Data are presented relative to the control group (mean ± SD) *: *p* ≤ 0.05; ** *p* ≤ 0.01, *** *p* value ≤ 0.001.

The antioxidant activity of GA, 3,4,5-THBS, and 3,4,5-TMBS was assessed in HIEC-6 cells using the fluorescent probe 2′,7′-dichlorodihydrofluorescein diacetate (H2DCFDA). This assay quantifies the oxidation of H2DCFDA to 2′,7′-dichlorofluorescein (DCF), which serves as a marker for intracellular reactive oxygen species (ROS) levels. Cells were exposed to compound concentrations ranging from 0.2 to 50 µM, and the resulting fluorescence intensities were compared to a control group treated with H2DCFDA alone. As shown in Figure 5, a significant reduction in ROS levels was observed in cells treated with GA and its derivatives compared to the control.

These results indicate that GA, THBS, and TMBS possess potent antioxidant activity, as demonstrated by their ability to lower ROS levels in treated cells effectively. This suggests that GA and its derivatives can protect HIEC-6 cells from oxidative stress, underscoring their potential as therapeutic agents against ROS-induced cellular damage.

### 3.5. Anti-Inflammatory Activity

**Figure 6:**
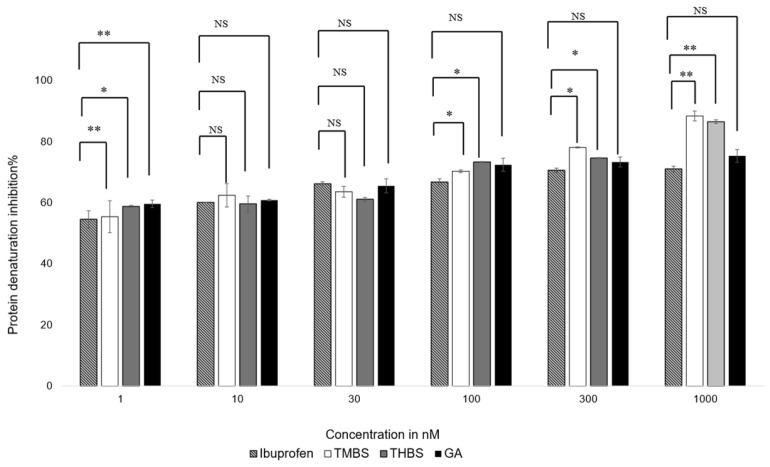
Protein denaturation inhibition % of ibuprofen (control), GA, 3,4,5-THBS, and 3,4,5-TMBS from 1 to 1000 nM using protein denaturation assay. Also, 100% inhibition of protein denaturation indicates the highest anti-inflammatory activity. NS: non-significant; *: *p* < 0.05; **: *p* < 0.01 (*n* = 3, mean ± SD).

The anti-inflammatory properties of GA, 3,4,5-THBS, and 3,4,5-TMBS were evaluated using a protein denaturation assay. The compounds were tested at concentrations ranging from 1 nM to 1000 nM, with ibuprofen included as a positive control. The results, illustrated in Figure 6, show the percentage inhibition of protein denaturation for each compound. The half-maximal effective concentration (EC50) values for TMBS, THBS, GA, and ibuprofen were calculated from dose–response curves and are summarized in Table 2.

All compounds demonstrated concentration-dependent anti-inflammatory activity. At concentrations below 30 nM, no significant differences in efficacy were observed among the compounds, except for 3,4,5-THBS, which showed a notable difference compared to ibuprofen. At 100 nM, 3,4,5-TMBS exhibited a significant increase in anti-inflammatory activity (*p* < 0.05), with further enhancement at higher concentrations. THBS displayed a similar trend, while GA did not show significant improvements in activity compared to ibuprofen at any concentration (*p* > 0.05). These findings suggest that 3,4,5-TMBS and 3,4,5-THBS have stronger anti-inflammatory effects than GA, particularly at higher concentrations.

### 3.6. COX-2 Activity Assay

**Figure 7:**
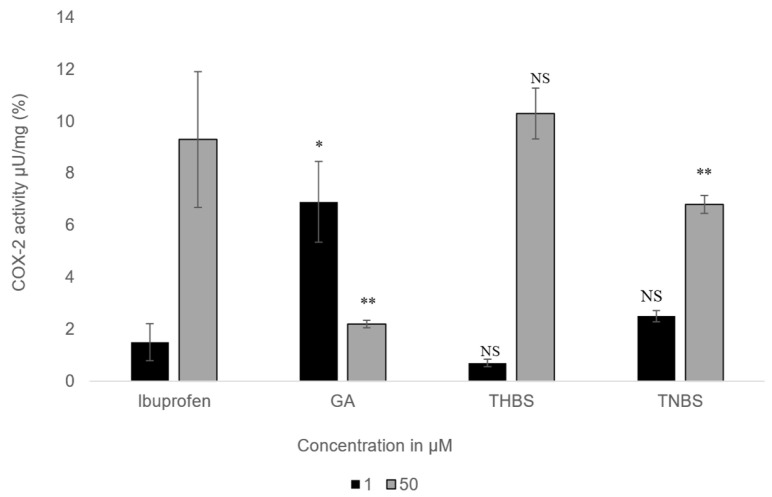
The percentage of COX-2 activity assessed for GA, 3,4,5-THBS, 3,4,5-TMBS, and ibuprofen (control) at two concentrations: 1 µM and 50 µM. (*n* = 3, mean ± SD). NS: non-significant; *p*-value > 0.05; *: *p* ≤ 0.05; ** *p* ≤ 0.01. COX-2 activity is measured as the amount of cyclooxygenase that generates 1.0 µmol of resorufin per minute at pH 7.4 and 25 °C.

The impact of GA, 3,4,5-THBS, and 3,4,5-TMBS on cyclooxygenase-2 (COX-2) activity was examined in HIEC-6 at two concentrations (1 µM and 50 µM). The results, presented in Figure 7, illustrate the percentage inhibition of COX-2 activity for each compound compared to ibuprofen, which served as the positive control.

At the lower concentration (1 µM), all compounds exhibited stronger COX-2 inhibition compared to the higher concentration (50 µM). 3,4,5-THBS showed inhibition levels similar to ibuprofen at both concentrations, with no statistically significant difference. 3,4,5-TMBS, however, displayed no significant difference in COX-2 inhibition relative to ibuprofen at 1 µM but demonstrated a highly significant increase in inhibition (*p* < 0.001) at 50 µM. GA, on the other hand, showed weaker COX-2 inhibition than ibuprofen at both concentrations, with its inhibitory effect being less pronounced at 50 µM. These findings suggest that 3,4,5-TMBS is particularly effective at higher concentrations, while 3,4,5-THBS maintains consistent inhibition comparable to ibuprofen. The results highlight the concentration-dependent variability in COX-2 inhibition among the tested compounds, with 3,4,5-TMBS emerging as a potent inhibitor at elevated concentrations.

### 3.7. CYP2D6 Activity

**Figure 8:**
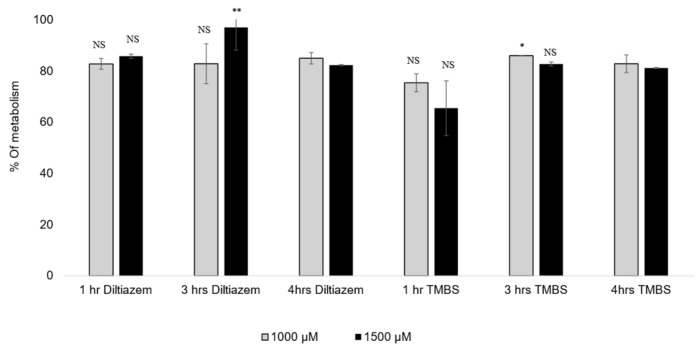
The % of diltiazem and 3,4,5-TMBS metabolized by CYP2D6 at three time intervals, 1, 3 and 4 h, at two concentrations, 1000 and 1500 µM. NS: non-significant; *: *p* < 0.05; **: *p* < 0.01 in comparison to Diltiazim (*n* = 3, mean ± SD).

The metabolization percentages of diltiazem and 3,4,5-TMBS by CYP2D6 at three distinct time intervals and two concentrations, specifically 1000 µM and 1500 µM, are illustrated in Table 3 and Figure 8. The data presented represent the average of two concentrations derived from three independent experiments. At a concentration of 1000 µM, an insignificant difference was observed in the percentage of 3,4,5-TMBS metabolized by CYP2D6 between the one-hour and four-hour time points. However, at a concentration of 1500 µM, a significant difference (*p* < 0.05) was noted, with the percentage of 3,4,5-TMBS metabolized rising from 65.5% to 81.2%.

**Figure 9:**
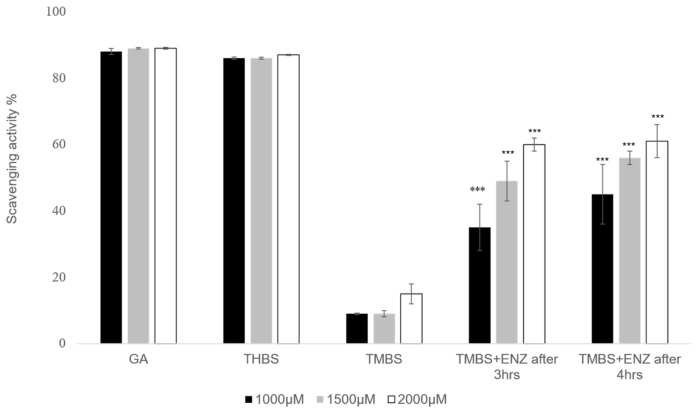
DPPH scavenging activity was repeated for GA (control), 3,4,5-THBS, 3,4,5-TMBS, and 3,4,5-TMBS with CYP2D6 enzyme after three hours (3,4,5-TMBS +ENZ after 3 h) and 3,4,5-TMBS with CYP2D6 enzyme after four hours (3,4,5-TMBS +ENZ after 4 h). ***: *p* ≤ 0.001 (*n* = 3, mean ± SD).

No significant differences were noted in the metabolism of 3,4,5-TMBS after 3 and 4 h across both concentrations tested. Likewise, the percentage of diltiazem metabolized at the 1 h and 3 h marks did not demonstrate a statistically significant difference (*p* > 0.05) when compared to the metabolism observed after 4 h. However, at a concentration of 1500 µM, the percentage of diltiazem metabolized after 3 h was significantly higher (*p* < 0.05) than that observed after 4 h. These results indicate that the interaction between the compounds and the enzyme CYP2D6 initiates within the first hour of the reaction, with the metabolic profiles of 3,4,5-TMBS and diltiazem being comparable at all tested concentrations and time intervals. To further assess the scavenging activity of 3,4,5-TMBS metabolites, an antioxidant assay was conducted after 3 and 4 h following the metabolism. After both 3 h and 4 h reaction periods, the enzyme was separated, and the resulting supernatant was utilized for the DPPH assay. Findings regarding the antioxidant activity of 3,4,5-TMBS, post-metabolism, at these time points, in comparison to GA and 3,4,5-THBS at the same concentration, are illustrated in Figure 9.

A notable increase in scavenging activity was observed at three and four hours post-reaction. Briefly, the percentage of scavenging activity for 3,4,5-TMBS at a concentration of 1000 µM was recorded as 35 % after three hours and increased to 45 % after four hours. Additionally, a higher concentration of 3,4,5-TMBS (2000 µM) demonstrated a further increase in scavenging activity, up to 61% after three and four hours.

### 3.8. Oral Bioavailability of GA, 3,4,5-THBS, and 3,4,5-TMBS

**Figure 10:**
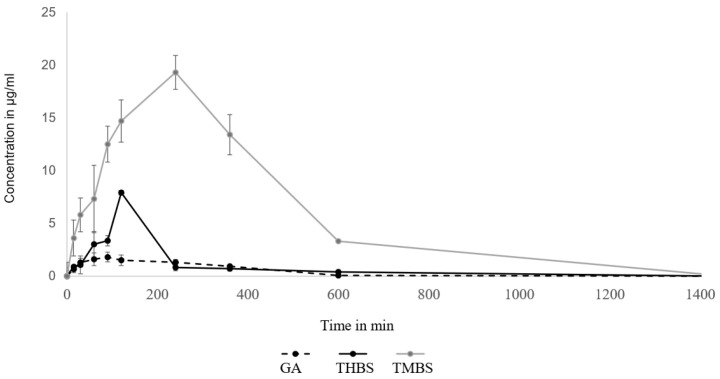
Oral bioavailability of GA, THBA, and 3,4,5-TMBS in rats after oral administration of 100 mg dose (*n* = 6, mean ± SD).

The pharmacokinetic parameters of GA were assessed utilizing a non-compartmental model, with the findings outlined in Table 3. The pharmacokinetics of GA, 3,4,5-TMBS, and 3,4,5-THBS in rat serum following an oral dose of 100 mg/kg were compared. The average serum concentration–time profiles of GA, 3,4,5-TMBS, and 3,4,5-THBS are illustrated in Figure 10. Key pharmacokinetic parameters were determined and presented in Table 4. Following oral administration, the time to reach maximum concentration (T_max_) was observed at 78 min for GA, 120 min for 3,4,5-THBS, and 172 min for 3,4,5-TMBS, with these differences considered statistically significant. Notably, the maximum concentration (C_max_) for 3,4,5-TMBS and 3,4,5-THBS was significantly greater than that of GA, indicating superior bioavailability and subsequent biodistribution for these compounds. The half-life (T_1/2_) in rats was recorded as 3.60 ± 0.94 h for GA, 2.10 ± 1.30 h for 3,4,5-THBS, and 7.17 ± 1.62 h for 3,4,5-TMBS, highlighting a significant difference for 3,4,5-TMBS and 3,4,5-THBS when compared to GA. Furthermore, the area under the curve from time zero to t (AUC0-t) for 3,4,5-THBS and 3,4,5-TMBS was substantially higher than that of GA, particularly for 3,4,5-TMBS, suggesting distinct pharmacokinetic profiles among the compounds. A marked increase in mean residence time (MRT) was noted for 3,4,5-THBS relative to GA, with values of 2.69 ± 4.53 and 7.66 ± 2.99, respectively, while 3,4,5-TMBS also exhibited a significant change compared to GA. The distribution volume at the terminal phase (Vz) for both 3,4,5-TMBS and 3,4,5-THBS was approximately ten-fold lower than that of GA.

It was worth noting that the apparent serum clearance CL/F, despite being insignificant in 3,4,5-THBS, was lower in 3,4,5-THBS and 3,4,5-TMBS (refer to Table 4).

## 4. Discussion

The antioxidant activity of gallic acid (GA) and its sulfonamide derivatives, 3,4,5-THBS and 3,4,5-TMBS, was evaluated using the DPPH radical scavenging assay. GA, a well-known polyphenol, has been extensively studied for its potent antioxidant properties, which are primarily attributed to the presence of three hydroxyl groups attached to its aromatic ring. These hydroxyl groups, particularly the one in the para position, facilitate intramolecular hydrogen bonding, enhancing its free radical scavenging capacity [12,31]. In line with this, our results demonstrated that GA and 3,4,5-THBS exhibited significant concentration-dependent antioxidant activity, whereas 3,4,5-TMBS showed negligible effects (*p* > 0.05). The EC_50_ values for GA and 3,4,5-THBS were approximately 30 µM and 27 µM, respectively, which are consistent with a previous study [32]. The lack of significant antioxidant activity in 3,4,5-TMBS may be attributed to the absence of hydroxyl groups, which are critical for radical scavenging. However, the methylation of phenolic compounds, as seen in 3,4,5-TMBS, has been reported to enhance antioxidant activity in certain contexts, suggesting that structural modifications can influence their biological efficacy [33]. The DPPH assay results were further corroborated by the reactive oxygen species (ROS) assay in HIEC-6 cells, where GA and its derivatives significantly reduced intracellular ROS levels. This aligns with the established role of GA as a non-enzymatic antioxidant capable of mitigating oxidative stress in cellular systems [34]. Interestingly, the metabolism of 3,4,5-TMBS by CYP2D6, an enzyme involved in O-demethylation, resulted in a marked increase in its antioxidant activity. This suggests that the metabolic activation of 3,4,5-TMBS may enhance its free radical scavenging potential, as evidenced by the increased DPPH scavenging activity observed after 3 and 4 h of enzymatic reaction (Figure 9). These findings highlight the potential of 3,4,5-TMBS as a prodrug that requires metabolic conversion to exert its antioxidant effects. In addition to their antioxidant properties, GA, 3,4,5-THBS, and 3,4,5-TMBS demonstrated significant anti-inflammatory activity, as assessed by the protein denaturation assay and COX-2 inhibition studies. The anti-inflammatory effects of these compounds were concentration-dependent, with 3,4,5-TMBS and 3,4,5-THBS showing comparable efficacy to ibuprofen, a standard anti-inflammatory agent. The phenol sulfonamide moiety present in 3,4,5-TMBS and 3,4,5-THBS has been previously reported to inhibit protein denaturation and suppress inflammatory pathways [31]. Furthermore, GA is known to exert anti-inflammatory effects through the modulation of mitogen-activated protein kinase (MAPK) and nuclear factor kappa B (NF-κB) signaling pathways, leading to reduced expression of inflammatory mediators such as cytokines, chemokines, and COX-2 [35,36,37]. Our findings indicate that 3,4,5-TMBS and 3,4,5-THBS not only retain the anti-inflammatory properties of GA but also exhibit enhanced COX-2 inhibition, making them promising candidates for further development as anti-inflammatory agents. The pharmacokinetic profiling of GA, 3,4,5-THBS, and 3,4,5-TMBS revealed distinct differences in their oral bioavailability and biodistribution. GA exhibited rapid absorption and elimination, with a T_max_ of 78 min and a half-life (t_1/2_) of 3.60 h, consistent with previous reports [30,38]. In contrast, 3,4,5-TMBS and 3,4,5-THBS demonstrated prolonged absorption and higher bioavailability, as evidenced by their significantly higher C_max_ and AUC_0–t_ values. The delayed T_max_ and extended half-life of 3,4,5-TMBS (7.17 h) suggest improved metabolic stability and sustained release, likely due to the sulfonamide group enhancing its passive diffusion across physiological membranes [39]. Additionally, the lower volume of distribution (Vz) and clearance (CL) of 3,4,5-TMBS and 3,4,5-THBS compared to GA indicate greater plasma retention and reduced extravascular distribution, which may contribute to their enhanced therapeutic efficacy. The structural modification of GA, particularly the replacement of hydroxyl groups with methoxy groups, has been shown to improve metabolic stability and membrane permeability [40], thereby enhancing oral bioavailability [38,41]. This is consistent with our observations, where 3,4,5-TMBS exhibited the highest bioavailability among the tested compounds. The increased AUC_0–t_ and AUC_0–∞_ values for 3,4,5-TMBS further underscore its potential for sustained therapeutic effects, making it a promising candidate for oral delivery. However, the distinct pharmacokinetic profiles of GA, 3,4,5-THBS, and 3,4,5-TMBS suggest that their absorption, distribution, and metabolism may vary significantly depending on physiological conditions, warranting further investigation.

While the findings highlight the potential of methoxylated flavones in cancer prevention, certain limitations must be acknowledged. Much of the evidence stems from in vitro and animal studies, which may not fully replicate human physiological conditions. Moreover, the bioavailability and metabolic behavior of these compounds in humans remain poorly understood, posing challenges for clinical application. Future studies should focus on clarifying the mechanisms behind their chemopreventive effects and exploring their interactions with other dietary components through rigorous clinical trials

## 5. Conclusions

The sulfonamide derivatives of gallic acid, 3,4,5-TMBS and 3,4,5-THBS, demonstrated antioxidant and scavenging activities comparable to that of GA at concentrations below 10 µM, suggesting that the sulfonamide modification of GA maintains its antioxidant effectiveness. Further methylation of 3,4,5-TMBS improved its pharmacokinetic properties, with superior metabolic stability and bioavailability compared to GA. When evaluated for anti-inflammatory activity, 3,4,5-TMBS and 3,4,5-THBS indicated enhanced effects, surpassing ibuprofen at higher concentrations, with 3,4,5-TMBS also displaying significant COX-2 inhibition at 50 µM. The pharmacokinetic analysis in Sprague Dawley rats revealed a longer half-life and higher bioavailability for 3,4,5-TMBS, suggesting improved systemic distribution and potential therapeutic advantages. These results support the idea that 3,4,5-TMBS and 3,4,5-THBS retain the beneficial antioxidant and anti-inflammatory properties of GA, while also exhibiting an improved pharmacokinetic profile. Accordingly, these sulfonamide derivatives may be considered a promising non-enzymatic antioxidant candidate for further research. As demonstrated in this study, their pharmacokinetic and biological profiles provide a solid foundation for their evaluation in preclinical and clinical settings, where their safety and therapeutic efficacy can be thoroughly investigated.

## Figures and Tables

**Table 1:** IC50 values of GA, 3,4,5-THBS, and 3,4,5-TMBS in HIEC-6 cells, as determined by the CCK-8 assay.

Compound	IC50 in µM
GA	47.55 ± 2.39
3,4,5-THBS	35.53 ± 1.52
3,4,5-TMBS	33.36 ± 0.70

**Table 2:** Protein denaturation EC50 values of ibuprofen (control), GA, 3,4,5-THBS, 3,4,5-TMBS (±SD).

Compound	EC50 in nM
Ibuprofen	0.13 ± 0.04
Gallic acid	0.62 ± 0.12
3,4,5-THBS	0.91 ± 0.02
3,4,5-TMBS	0.691 ± 0.09

**Table 3:** Metabolism of diltiazem and 3,4,5-TMBS at three time points 1 h, 3 h, and 4 h, at two concentrations 1000 µM, and 1500 µM (*n* = 3, mean ± SD).

Concentration in µm	1 h	3 h	4 h
Diltiazem 1000	827.81 ± 21.72	828.85 ± 78.11	849.914 ± 0.62
Diltiazem 1500	1286.97 ± 12.84	1455.72 ± 133.01	1234.57 ± 6.02
3,4,5-TMBS 1000	754.16 ± 35.45	860.62 ± 0.71	832.9 ± 54.94
3,4,5-TMBS 1500	983.79 ± 161.19	1240.53 ± 12.19	1248.09 ± 5.02

**Table 4:** This table summarizes the pharmacokinetic parameters of GA, 3,4,5-TMBS, and 3,4,5-THBS in rats after the oral administration of a 100 mg dose (*n* = 6, mean ± SD). Values are classified as non-significant (NS) where *p* > 0.05, significant (*) where *p* ≤ 0.05, high significance (**) for *p* ≤ 0.01, and very high significance (***) for *p* ≤ 0.001.

Parameter	Unit	GA	3,4,5-THBS	3,4,5-TMBS
C_max_	μg/mL	2.14 ± 0.88	9.56 ± 1.90 ***	25.76 ± 6.03 ***
t_1/2_	h	3.60 ± 0.94	2.10 ± 1.30 ^NS^	7.17 ± 1.62 **
T_max_	h	1.30 ± 0.22	2.0 ± 0.10 ***	2.875 ± 1.31 *
AUC_0–t_	μg/mL × h	9.32 ± 4.61	24.15 ± 0.27 *	172.77 ± 49.28 ***
AUC_0–∞_	μg/mL × h	12.96 ± 5.62	24.22 ± 7.37 ^NS^	386.35 ± 43.81 ***
MRT_0–∞_	h	7.66 ± 2.99	2.69 ± 0.36 *	11.88 ± 0.61 *
V_z_	(mg)/(μg/mL)	55.46 ± 23.99	7.39 ± 2.16 ***	3.20 ± 0.55 ***
Cl/f	(mg)/(μg/mL)/h	8.85 ± 3.37	4.39 ± 1.30 ^NS^	0.37 ± 0.22 *

The maximum observed concentration (C_max_); the time where the maximum observed concentration is detected (T_max_); the half-life of the drug (t_1/2_); the area under the zero and first-moment curves (AUC_0–t_); the area under the zero sampling time to infinity (AUC_0_-∞); the mean resistance time (MRT); the volume of distribution based on the terminal slope (V_z_); clearance (Cl/f).

## Data Availability

All data is contained within the article.
